# Gut microbiota analysis for prediction of clinical relapse in Crohn’s disease

**DOI:** 10.1038/s41598-022-23757-x

**Published:** 2022-11-19

**Authors:** Sylvie Buffet-Bataillon, Guillaume Bouguen, François Fleury, Vincent Cattoir, Yann Le Cunff

**Affiliations:** 1 INSERM, Institut NUMECAN (Nutrition Metabolisms and Cancer), CHU Rennes, Université Rennes 1, 35000 Rennes, France; 2 CIC 1414, INSERM, Institut NUMECAN (Nutrition Metabolisms and Cancer), CHU Rennes, Université Rennes 1, 35000 Rennes, France; 3 U1230, INSERM, CHU Rennes, Université Rennes 1, 35000 Rennes, France; 4 Dyliss - Dynamics, Logics and Inference for biological Systems and Sequences, Inria Rennes – Bretagne Atlantique, Université Rennes 1, Rennes, France

**Keywords:** Crohn's disease, Computer science

## Abstract

The role of intestinal bacterial microbiota has been described as key in the pathophysiology of Crohn’s disease (CD). CD is characterized by frequent relapses after periods of remission which are not entirely understood. In this paper, we investigate whether the heterogeneity in microbiota profiles in CD patients could be a suitable predictor for these relapses. This prospective observational study involved 259 CD patients, in which 41 provided an additional total of 62 consecutive fecal samples, with an average interval of 25 weeks in between each of these samples. Fecal microbiota was analyzed by massive genomic sequencing through 16 S rRNA amplicon sampling. We found that our 259 CD patients could be split into three distinct subgroups of microbiota (G1, G2, G3). From G1 to G3, we noticed a progressive decrease in alpha diversity (p ≤ 0.0001) but no change in the fecal calprotectin (FC) level. Focusing on the 103 consecutive samples from 41 CD patients, we showed that the patients microbiota profiles were remarkably stable over time and associated with increasing symptom severity. Investigating further this microbiota/severity association revealed that the first signs of aggravation are (1) a loss of the main anti-inflammatory Short-Chain Fatty Acids (SCFAs) *Roseburia, Eubacterium*, *Subdoligranumum*, *Ruminococcus* (P < 0.05), (2) an increase in pro-inflammatory pathogens *Proteus*, *Finegoldia* (P < 0.05) while (3) an increase of other minor SCFA producers such as *Ezakiella*, *Anaerococcus, Megasphaera, Anaeroglobus*, *Fenollaria* (P < 0.05). Further aggravation of clinical signs is significantly linked to the subsequent loss of these minor SCFAs species and to an increase in other proinflammatory Proteobacteria such as *Klebsiella, Pseudomonas, Salmonella, Acinetobacter, Hafnia* and proinflammatory Firmicutes such as *Staphylococcus, Enterococcus, Streptococcus.* (P < 0.05). To our knowledge, this is the first study (1) specifically identifying subgroups of microbiota profiles in CD patients, (2) relating these groups to the evolution of symptoms over time and (3) showing a two-step process in CD symptoms’ worsening. This paves the way towards a better understanding of patient-to-patient heterogeneity, as well as providing early warning signals of future aggravation of the symptoms and eventually adapting empirically treatments.

## Introduction

Crohn’s disease (CD) is characterized by chronic inflammation of the gastrointestinal (GI) tract, which involves complex interactions between the host immune system, intestinal mucosa and gut microbiota^1^. Numerous studies have found an imbalance in the gut microbiota of CD patients compared to non-CD controls with an overall loss of diversity, a depletion of firmicutes and an increase in Proteobacteria^2–4^. The chronic, unpredictable nature of the disease and its debilitating effect on all aspects of life are major concerns for patients with CD with a broad diversity of symptoms^5,6^. For instance, 21–47% of CD patients present in addition to digestive symptoms, systemic and extra-intestinal manifestations^7^. Half of CD patients develop intestinal complications, such as strictures or fistulae, within 10 years of diagnosis and require surgery in the 20 years following the diagnosis^8,9^. In a nutshell, it is well documented that CD displays a large heterogeneity of symptoms between patients—from mild to heavily impairing everyday life -, as well as in time with a relapsing–remitting dynamics that is also yet to be fully understood. It is also still unclear how such inter-patient heterogeneity in symptoms and CD evolution is associated with microbiota heterogeneity. In this paper, we harness a novel large longitudinal cohort of Crohn’s patients to unravel this relationship.

We thus investigated (1) the heterogeneity of gut microbiota in CD patients, (2) for the first time the temporal evolution of the disease in order to link symptoms’ evolution and microbiota composition, and (3) the “key microbial signatures” during the transitions from one microbiota profile to another in order to pave the way towards personalized diagnosis and treatment of CD.

## Methods

### Cohort of patients

A total of 259 patients with CD were included at the referral center Hospital University of Rennes (France) during a two-year period and provided informed consent for this observational, non-interventional study. Patients were informed to be included in a prospective research database (Rennes, approved by the Commission Nationale Informatique et Liberté (CNIL) No. 1412467) which allowed us to perform this study without additional consent as the data were retrieved from the standard follow-up of patients.

Inclusion criteria were CD patients diagnosed using standard endoscopic, histological or radiological criteria, aged from 16 to 80 years old. Exclusion criteria were patients with ulcerative colitis, and/or with a stoma. Details regarding age, female sex, smoking, gastrointestinal surgery, Montreal classification for CD (A, B, L), fecal calprotectin (FC)(µg/g), treatment (Anti-TNF, Thiopurine-methotrexate (MTX), Antibiotics) were collected on the same day of fecal samples and are shown in Table 1.

**Table 1:** Characteristics of the cohort (n = 259 patients/n = 41 patients).

	CD patients [(n = 259)/(n = 41)]
Age (mean ± SD)	41 ± 15/45 ± 15
Female sex (%)	145 (56%)/20 (49%)
Smoking (%)	43 (25%)/9 (22%)
Gastrointestinal surgery	54 (21%)/14 (34%)
Montreal A (%)	A1: 35 (19%)/4 (10%); A2: 124 (68%)/30 (77%); A3: 23 (13%)/5 (13%)
Montreal B (%)	B1: 111 (62%)/23 (61%); B2: 39 (22%)/10 (26%); B3: 29 (16%)/5 (13%)
Montreal L (%)	L1: 54 (29%)/10 (25%); L2: 31(17%)/7 (18%); L3: 99(54%)/23 (57%)
Fecal calprotectin (μg/g), median (IQR)	122.5 (31.75–529.75)/322 (72–531.5)
Anti-TNF (%)	154 (59%)/34 (83%)
Thiopurines (%)	126 (48%)/28 (68%)
MTX (%)	141 (54%)/8 (20%)

Montreal A (age at diagnosis): A1: < 16 years; A2: 17–40 years; and A3: > 40 years.

Montreal L (disease location): L1 ileum; L2 colon; L3 ileum–colon.

Montreal B (disease behavior): B1 inflammatory; B2 structuring; B3 penetrating.

Anti-TNF: infliximab, adalimumab, ustekinumab, vedolizumab.

Thiopurines: azathioprine, 6-mercaptopurine.

*CD* Crohn’s disease, *MTX* methotrexate.

### Patient selection

A subgroup of 41 patients from the cohort had several temporal fecal samplings over the longitudinal study (n = 103 samples in total). The Harvey-Bradshaw Index (HBI) was assessed to describe the status of a patient at the time when the sample was taken. HBI thresholds were used to define three groups “Remission” (HBI < 5 with abdominal pain = 0 and complications = 0) (n = 42); “Mild-Moderate” (HBI = 5–8 with abdominal pain = 1 (mild) or 2 (moderate) and complications = 0) (n = 28); and “Severe” (HBI > 8 with abdominal pain = 3 and at least one complications) (n = 33)^10^.

### Gut microbiota analysis

We used a standard procedure for fecal microbiota profiling using 16S rRNA-based metagenomic sequencing and bioinformatic analysis. According to the International Human Microbiome Standards (IHMS), patients were asked to collect fecal samples immediately after defecation in a sterile container (from VWR).Then, the stool samples were stored up to 24 h at 4 °C. When received at the laboratory, all samples were manually homogenized and weighed into separate aliquots for storage at 80 °C until DNA extraction.

The extracted DNA was used for the assessment of the microbial populations using 16S rRNA gene sequence-based microbiota analysis. DNA was extracted from the fecal specimens with the automated MagNA Pure system (Roche Diagnostics GmbH, Mannheim, Germany), using the MagNA Pure LC DNA Kit III (bacteria, fungi) (Roche) according to the manufacturer's recommendations. Pre-isolation steps as mechanical lysis (30 s at 6000 rpm) on the MagNa Lyser Instrument, and heat lysis (10 min at + 65 °C and at 95 °C) were added to perform DNA isolations.

We followed the steps described in the Illumina 16S sample preparation guide to amplify the V3 and V4 region, add Illumina sequencing adapters and dual index barcodes to the amplicon target.

Primers were designed according to the V3-V4 regions of bacteria (the upstream primer: PCR_341 F is 5'-CCTACGGGNGGCWGCAG-3', and the downstream primer: PCR_785R is 5'-GACTACHVGGGTATCTAATCC-3')^11^. The Illumina overhang adapter sequences used were as follows: Forward overhang: 5’ TCGTCGGCAGCGTCAGATGTGTATAAGAGACAG-[PCR_341F]; Reverse overhang: 5’ GTCTCGTGGGCTCGGAGATGTGTATAAGAGACAG-[PCR_785R].

Primers linked to adapters were used for PCR amplification; the PCR products were purified, quantified, and then normalized to form a sequencing library. These were sequenced by Illumina MiSeq as outlined in the Illumina 16S sample preparation guide.

The individual sequence reads obtained were filtered, trimmed and processed as described by Escudie and al. according to Find Rapidly OTUs with Galaxy Solution (FROGS)^12^. All reads were classified to the lowest possible taxonomic level (species or genus) using FROGS. The SILVA database used for taxonomic assignment was SILVA 16S v132 A table of 16S rRNA Operational Taxonomic Units (OTUs) was then generated.

### Statistical analysis

For the clustering of microbiota profiles, we performed the following procedure. First, the clr transform was applied to the OTU count table (patients in row, OTU labels in column) from the R phyloseq package (v1.32.0), similar to Tsilimigras et al.^13^. This table was then normalized to achieve a mean of 0 and a standard deviation of 1 per column, akin to the first step of performing a Principal Component Analysis (PCA)^14^. The non-supervised clustering used is an Ascending Hierarchical Clustering, with a euclidean distance and the Ward aggregation method (sciPy Python package, v1.8.1)^15^. As we opted for a non-supervised approach, we did not use a train/test split, which would have been required if we had to train a model to predict the severity of the symptoms. The PCA analysis and the Silhouette computation to assess the number of clusters were performed in Python with the package scikit-learn (sklearn). The highest silhouette coefficient identified two distinct groups and the second highest identified three groups.

To assess alpha diversity, the Chao1 and Shannon diversity indices were used. To investigate whether dysbiosis could be linked to inflammation, we tested whether fecal calprotectin levels would vary between groups of microbiota, using a Kruskal–Wallis test. Then, to estimate the temporal evolution of dysbiosis, groups identified by hierarchical clustering were assessed with respect to symptoms’ severity using Fisher's exact test. Finally, to identify the “key microbial signatures'' in the dynamics of the disease, the differential abundance of species between patients was determined using DESeq2 and apglm packages with p-value adjusted for multiple testing using the Benjamini–Hochberg method (cut-off = adjusted p < 0.05). Since confounding factors may influence the fecal microbiome^7,16,17^ and to feel confident in the relevant changes in bacterial groups observed in our study, we also quantified the influence of known factors as smoking and disease localization on the three groups of microbiota. Statistical analysis and data visualizations were performed using R (v.3.5.1) with multiple packages, including ‘phyloseq’ (v1.32.0), ‘philr’ (v1.14.0), ‘ggplot 2’ (v3.3.6), ‘grid’ (v4.1.3), ‘ape’ (v.4.1), ‘scales’ (v1.2.0), ‘DESeq 2’ (v1.28.1) and ’apglm’ ( v1.1.16 from BiocManager v1.30.18).

### Ethics approval and consent to participate

The study was carried out in accordance with national guidelines and authority regulations for research. All experimental protocols were approved by a named institutional review board and/or licensing ethics committee. Patients were informed to be included in a prospective research database (Rennes, Commission Nationale Informatique et Liberté (CNIL) No. 1412467) that allowed us to perform study without additional consent as the data were retrieved from the standard of care follow-up of patients.

## Results

### Characteristics of CD patients

The characteristics of the 259 patients and 41 patients with CD are summarized in Table 1. More than 80% of the cohort displays inflammation in the lower part of the gut. Only two of the 259 patients (0.08%) received antibiotics (metronidazole or ciprofloxacin) within 90 days of microbiota analysis. As the data retrieved from the standard follow-up of patients, the timing between consecutive samples and the total number of samples collected differed between the 41 patients. Precisely, 26 patients had 2 samples, 9 patients had 3 and 6 patients had 4. The median time interval between two samples was 15 weeks and the average time interval was 25 weeks.

### Three distinct groups of microbiota profiles in CD patients

**Figure 1:**
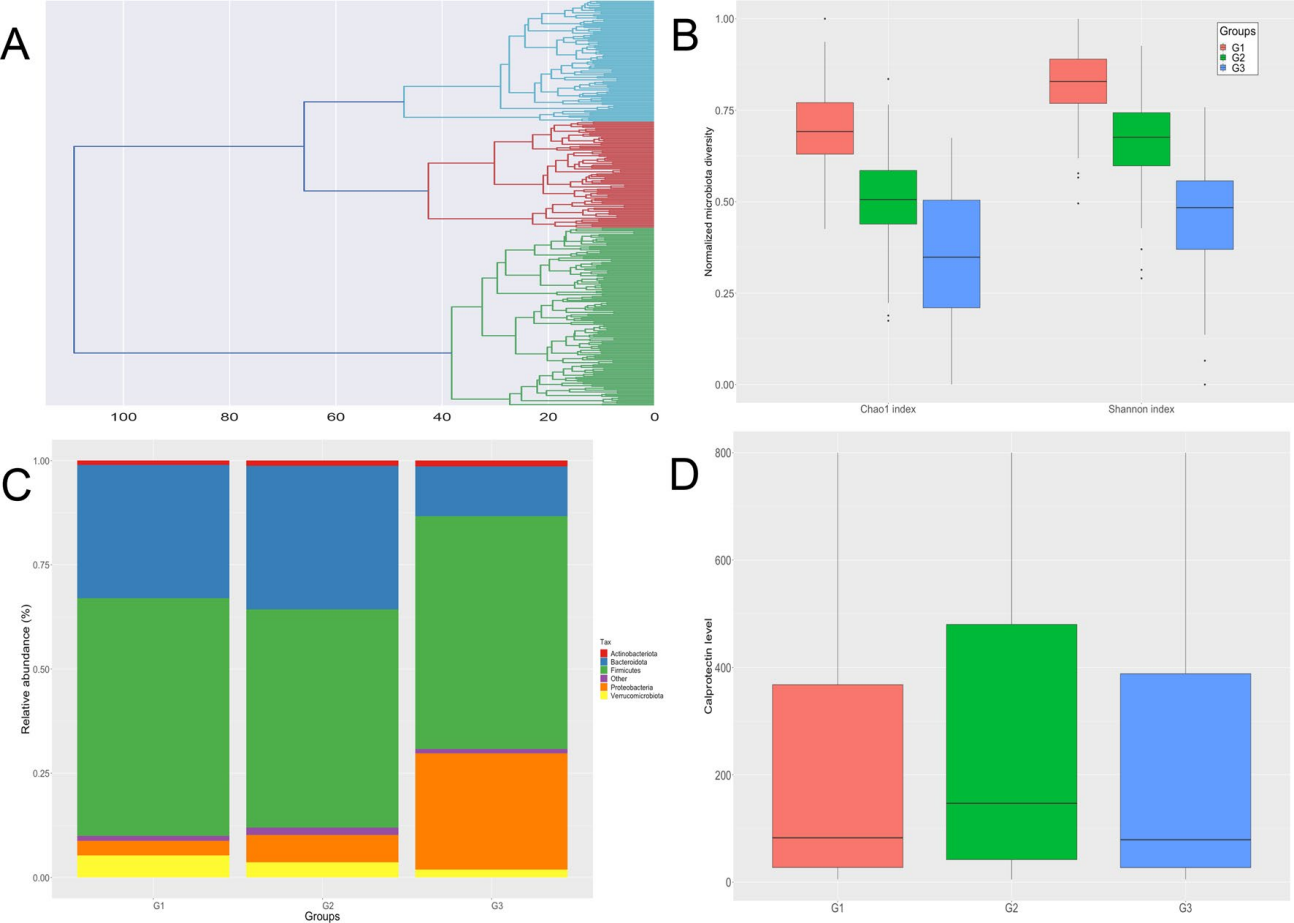
Gut microbiota from 259 CD patients (n = 321 samples). (**A**) Ascending hierarchical classification (AHC) of the 321gut microbiota : three distinct groups (G1, G2, G3) are identified, depicted in blue, red and green, respectively. (**B**) A decreasing Shannon index and Chao1 index is observed from G1 to G3 (p = 2.6e−15 and 4.02e−13 respectively). (**C**) Global composition of gut microbiota at the phyla level in G1, G2 and G3. (**D**) Association between the three groups of gut microbiota G1, G2 and G3 and Calprotectin levels in CD patients: Calprotectin levels are not significantly different between G1, G2 and G3, despite differences in degree of dysbiosis.

Of the 321 gut microbiota samples analyzed from 259 patients, our clustering method identified three groups of microbiota profiles (G1, G2 and G3) that significantly differed in terms of gut microbiota (Fig. 1A). Species diversity was found to decrease from G1 to G2 and from G2 to G3, based on Chao1 and Shannon indexes (Fig. 1B) (Kruskal–Wallis test, p = 4.02e−13 and 2.6e−15, respectively). The analysis of the gut microbiota showed significant changes in the proportion of the microbial phyla Proteobacteria between G1, G2 and G3 groups (Fig. 1C) (Fisher exact test, p < 0.05). No association between confounding factors such as smoking or disease localization and the three groups of microbiota was found. Interestingly, while the three groups displayed a broad diversity of microbiota, they did not display significant differences in terms of calprotectin levels (Fig. 1D, Kruskal–Wallis test, p = 0.26).

### Dynamics of fecal bacterial clustering in CD Patients over time

**Figure 2:**
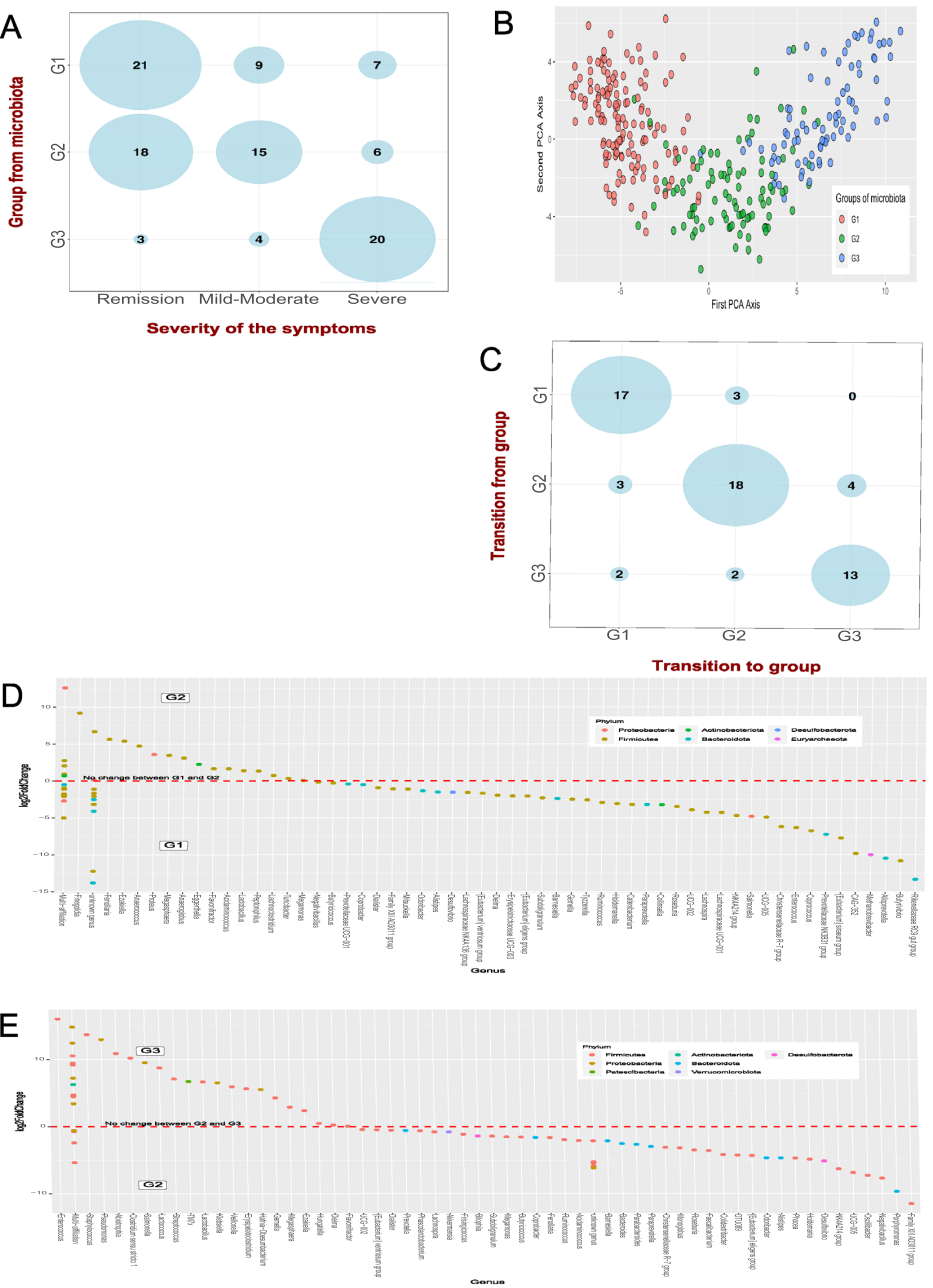
Gut microbiota from 41 CD patients (n = 103 several consecutive samples). (**A**) Association between the groups of gut microbiota G1, G2 and G3 and severity of the symptoms in CD patients. From the groups G1 to G3, an increase in symptoms severity is observed (p = 3.46e−6). (**B**) Projection of the three groups of gut microbiota G1, G2 and G3 with principal component analysis (PCA): G1, G2 and G3 are represented by three different colors. (**C**) Transition of the three groups of gut microbiota G1, G2 and G3 between two consecutive samples: overall, CD patients remain in the same group over time. (**D**) Significant (p < 0.05) log-fold changes in the abundances of bacterial species in G2 compared to G1. Positive log-fold change points out an increase in abundance in G2 compared to G1, while negative log-fold change points out a reduction in abundance. (**E**) Significant (p < 0.05) log-fold changes in the abundances of bacterial species in G3 compared to G2. Positive log-fold change points out an increase in abundance in G3 compared to G2, while negative log-fold change points out a reduction in abundance.

We then focused on the subset of 41 patients with multiple microbiota samples (from 2 to 4 samples per individual), which represented 103 samples overall. In Fig. 2A, we show a strong association between microbiota composition and symptoms severity (Fisher’s exact test, p = 3.46e−06): G3 was mainly associated to “Severe” CD cases [20/27 (74%)]; G2 to “Remission” CD cases [18/39 (46.1%)] as well as to “Mild-Moderate” CD cases [15/39 (38.5%)]; and finally, G1 to “Remission” CD cases [21/37 (56%)]. Figure 2B displays the three groups in the Principal Component Analysis (PCA) plan, that is, a 2D projection of the patients’ microbiota. One can notice that G2 is positioned between G1 and G3, in agreement with the increase of symptoms severity from G1 to G3. In Fig. 2C, we show the transitions between groups from two successive medical appointments. No transitions from G1 to G3 and only two transitions from G3 to G1 were observed. Moreover, every observed improvement in terms of symptoms was associated with either a stability of the microbiota group or a transition either from G3 to G2 or from G2 to G1 (n = 15/15). As such, our results hint that the more common transitions from G1 to G2 or from G2 to G3 could be key indicators of the disease's evolution over time.

### Key microbial signatures to predict changes in CD symptoms over time

We then investigated in more detail the “key microbial signatures” i.e. the difference between G1, G2 and G3 microbiota profiles, as they result in different dynamics of the disease.

Figure 2D shows that the first sign of aggravation (transition from G1 to G2) is threefold: (1) a decrease of the main anti-inflammatory microorganisms belonging to the main SCFA-producing bacteria (*Roseburia, Eubacterium*, *Subdoligranumum*, *Ruminococcus*) (p < 0.05), (2) an increase in pro-inflammatory pathogens (*Proteus*, *Finegoldia*) (p < 0.05) and (3) an increase of minor SCFA producers (*Ezakiella*, *Anaerococcus, Megasphaera, Anaeroglobus*, *Fenollaria*) (p < 0.05). Further aggravation of clinical signs (transition from G2 to G3) (Fig. 2E) is significantly linked (1) to a deeper loss of the minor SCFA-producing bacteria (p < 0.05) and (2) to an increase in other pro-inflammatory Proteobacteria (such as *Klebsiella, Pseudomonas, Salmonella, Acinetobacter, Hafnia*) and pro-inflammatory Firmicutes (such as *Staphylococcus, Enterococcus, Streptococcus*) (p < 0.05).

## Discussion

In this study, we focused on characterizing the microbiota heterogeneity in CD patients in order to better understand the clinical evolution of the disease, rather than the traditional comparison between CD and controls or between CD and other IBDs^18–25^. We also took advantage of longitudinal information. Indeed, it has been shown that longitudinal profiling of multi-omics datasets even from smaller cohorts have higher performance and information richness than larger cohorts without longitudinal profiling. This has been demonstrated in other complex diseases such as diabetes and obesity^26,27^. Finally, we decided to take advantage of non-supervised clustering techniques of microbiota in order to shed light on inter-patient heterogeneity, as advocated in recent reviews concerning microbiota analysis^14,28,29^.

We showed that CD is characterized by three groups of microbiota profiles, G1 being akin to normobiosis in terms of species diversity while G2 and G3 display some form of dysbiosis. In agreement with others, dysbiosis was characterized by a marked reduction in bacteria belonging to the SCFA-producing bacteria of the phylum Firmicutes and an increased presence of the phylum Proteobacteria^19,22,30–33^.

In terms of markers of dysbiosis, FC is considered as the standard non-invasive marker for assessing disease activity in CD and has been shown to have clinical utility especially in monitoring disease activity, relapse, response to therapy and patient-reported outcomes in patients with CD^34–36^. Thus, we tried to find an association between FC and dysbiosis. As previously described in another study^37^, and in agreement with a recent study^38^, we showed no link between the severity of dysbiosis and FC levels.

We therefore focused on microbiota as a putative marker for CD symptom severity. To our knowledge, our study is the first to link a progressive change of microbiota in CD to a worsening of the CD symptoms. The results of the PCA confirmed the idea of a progression of the disease from G1 to G3 and the first axis of variability might very well be considered as a novel informative “score” for the severity of the disease that is worth computing in medical studies.

It is well established that dysbiosis is key to CD. Here we found that there were “key signatures'' of symptoms’ worsening. The first transition from G1 to G2 that triggers symptoms severity is characterized by the loss of the main anti-inflammatory SCFA producers (*Roseburia, Eubacterium*, *Subdoligranumum*, *Ruminococcus*) and increased pro-inflammatory bacteria such as *Proteus* and *Finegoldia*. These findings are in accordance with Neumann et al. and Zhang et al.^39,40^. Neumann et al. showed for the first time *Finegoldia* as an inducer of inflammation due to the interaction with human neutrophils^39^. *Proteus* has been recently shown to be a key factor in predicting disease relapse^40^. The depletion of SCFA-producing bacteria is interesting in the context of these symptoms-microbiota interaction and the natural history of CD. Indeed, it has been shown that SCFAs promote anti-inflammatory T and B cell responses as well as an anti-inflammatory phenotype of intestinal macrophages^41–46^. Finally, previous studies also showed that SCFAs/butyrate have inhibitory effects on inflammatory response, contributing to intestinal homeostasis and cancer protection^47–51^.

Then, the second transition from G2 to G3, associated to a further worsening of the symptoms’ severity, is characterized by the increase of others pro-inflammatory bacteria (*Klebsiella, Pseudomonas, Salmonella, Acinetobacter, Hafnia*, *Staphylococcus, Enterococcus, Streptococcus*). With Quantitative Microbiome Profiling, in agreement with our study, Vieira-Silva et al. have also identified the same genera *Enterococcus*, *Escherichia*, *Fusobacterium*, *Streptococcus*, and *Veillonella* as biomarkers for clinical severity^52^. Precisely, *Fusobacterium* and *Veillonella* were associated in patients with higher gastrointestinal inflammation. *Enterococcus* was particularly linked to biliary obstruction severity.

The challenge remains to understand whether existing or future treatments might be able to stop or reverse dysbiosis progression, improving the prognosis and changing the natural history of CD. We believe that the knowledge of the sequence in microbiota changes we identified in this paper might provide further insight into personalized fecal microbiota transplantation (FMT) by providing a step-by-step remediation process. Indeed, in a very recent study, (FMT) was effective to maintain remission in CD patients^53^. This link between microbiota and CD symptoms also strengthens the growing efforts regarding pre- and probiotic therapies. More specifically, our results suggest that SCFA production is associated with a restoration of intestinal homeostasis and sustained remission in CD patients. So far, probiotic treatments have not shown a significant effect in inducing or maintaining remission of active or quiescent CD, or in preventing relapse of CD after surgically-induced remission^54,55^. However, probiotics evaluated in these studies were not SCFA-producing bacteria.

Interestingly, a recent study provided proof-of-concept evidence for the therapeutic potential of SCFAs-producing bacteria in CD. In CD patients, treatment with a mix of 6 SCFAs-producers (*Butyricicoccus pullicaecorum 25-3T, Faecalibacterium prausnitzii, Roseburia hominis, Roseburia inulinivorans, Anaerostipescaccae, and Eubacterium hallii*) improved butyrate production, colonization capacity in mucus and lumen-associated CD microbiota as well as epithelial barrier integrity^56^. Such approaches may efficiently complement anti-TNFα therapy for reconstituting a healthy microbiome.

## Conclusions

The purpose of the study was to better understand disease history, heterogeneity and to show the potential for machine learning to assist clinicians with personalized CD treatment^57^. Our study confirms that unsupervised machine learning approaches are suited to characterize the gut dysbiosis and shows the association between this dysbiosis and symptom severity. Dysbiosis degree should be assessed along CD history to optimize CD management.

## Data Availability

The datasets analysed during the current study are publicly available: https://doi.org/10.57745/CMF9FC.
